# Real-world evidence on predictors of antimicrobial resistance among patients with chronic liver disease

**DOI:** 10.3389/fmed.2026.1799975

**Published:** 2026-04-13

**Authors:** Geetha Kandasamy, Lavanya Selvaraj, Khalid Orayj, Mona Almanasef, Rayah Asiri, Asma Alzaydani, Rahaf Abdullah Assiri, Divyadarshini Srikanth, Jeevan Krishnayya Ravikumar

**Affiliations:** 1 Department of Clinical Pharmacy, College of Pharmacy, King Khalid University, Abha, Saudi Arabia; 2 Department of Pharmacy Practice, PSG College of Pharmacy, Peelamedu, Coimbatore, India; 3 Department of Pharmacy, Rijal Almaa General Hospital, Ministry of Health (MOH), Rijal Almaa, Aseer, Saudi Arabia; 4 Pharmacy Department, Aseer Central Hospital Abha, Abha, Saudi Arabia

**Keywords:** antibiotic susceptibility, antimicrobial resistance, chronic liver disease, gram-negative bacteria, gram-positive bacteria, multidrug resistance, predictors, real-world evidence

## Abstract

**Introduction:**

Chronic liver disease (CLD) predisposes patients to bacterial infections, with multidrug-resistant (MDR) pathogens posing significant challenges to clinical management and outcomes. Understanding the prevalence, resistance patterns, and predictors of MDR is essential for optimizing antimicrobial therapy. This study aimed to generate real-world evidence on the clinical, demographic, and healthcare-related predictors of antimicrobial resistance and to characterize local pathogen distribution and resistance patterns among patients with CLD in a tertiary care setting.

**Methods:**

This retrospective observational cohort study (February–August 2024) included adult patients with CLD and culture-positive bacterial infections from tertiary care hospitals in Coimbatore, India. Antimicrobial susceptibility patterns, including MIC50, MIC90, and trends, were analyzed using WHONET v5.6. Categorical and continuous variables were compared using Chi-square and Mann–Whitney *U*-tests, respectively. Predictors of MDR infection were identified using logistic regression analysis.

**Results:**

Among 317 patients with CLD, the mean age was 50.9 ± 10.4 years (median 50, IQR 44-59), and 83.9% were male. Single-organism infections accounted for 76.0%, and 56.5% were community-acquired. *Escherichia coli* (37.5%) and *Klebsiella pneumoniae* (14.5%) were the most frequently isolated pathogens, with high MDR rates (78.2% and 82.6%, respectively). Both organisms were significantly associated with increased odds of MDR (*E. coli*: OR 2.41, 95% CI 1.45-4.01; *K. pneumoniae*: OR 3.12, 95% CI 1.58–6.15; *p* < 0.001). MDR prevalence was higher among Gram-negative isolates compared with Gram-positive isolates (70.2 vs. 51.9%; RR 1.35, 95% CI 1.07–1.70; *p* = 0.003). Gram-positive isolates remained largely susceptible to glycopeptides, oxazolidinones, and tetracyclines, while tigecycline (79.1%; *p*-trend = 0.041) and aminoglycosides (60.3%; *p*-trend = 0.019) retained activity against Gram-negative isolates. Independent predictors of MDR infection included age >60 years (adjusted OR 1.89), male gender (adjusted OR 1.67), healthcare-associated infection (adjusted OR 3.14), Gram-negative etiology (adjusted OR 2.73), and prior antibiotic exposure (adjusted OR 2.98; all *p* < 0.05).

**Discussion:**

MDR infections are highly prevalent among patients with CLD, predominantly driven by Gram-negative pathogens such as *Escherichia coli* and *Klebsiella pneumoniae*. Resistance was more common in healthcare-associated infections and was characterized by reduced susceptibility to carbapenems, cephalosporins, and fluoroquinolones. Tigecycline and aminoglycosides retained relatively preserved activity. Key predictors of MDR included older age, male gender, Gram-negative etiology, healthcare exposure, and prior antibiotic use, underscoring the need for targeted antimicrobial stewardship strategies.

## 1 Introduction

Antimicrobial resistance (AMR) represents a major global public health concern, contributing to an estimated 4.95 million deaths worldwide in 2019, including 1.27 million deaths directly attributable to resistant infections (1). If current trends continue, AMR is projected to result in up to 10 million deaths annually and an economic burden of USD 100 trillion by 2050 (2). Currently, over 700,000 deaths each year are directly linked to infections caused by multidrug-resistant organisms (MDROs) (2, 3). AMR poses particular challenges for vulnerable patient populations, where early identification of resistance predictors may support timely antimicrobial selection and inform management strategies (4).

Patients with chronic liver disease (CLD), particularly those with cirrhosis, are disproportionately affected by bacterial infections and AMR. Cirrhosis is associated with systemic inflammation, immune dysregulation, and increased intestinal permeability, increasing susceptibility to secondary infections (5, 6). Bacterial infections frequently contribute to acute-on-chronic liver failure (ACLF), a condition associated with elevated short-term mortality (7). Infections occur predominantly in advanced cirrhosis, as classified by the Child–Pugh score, with prevalence ranging from 25% to 46% and recurrent episodes reported in nearly half of affected patients (8–12).

Bacterial infections in CLD are associated with accelerated disease progression and higher mortality, irrespective of baseline severity (2, 3, 6, 13, 14). Long-term outcomes are poorer in cirrhotic patients experiencing bacterial complications, with 30-month survival rates reported at 34% vs. 62% in patients without infection (15–18). Mortality is further increased when infections involve multidrug-resistant (MDR) bacteria, defined as organisms exhibiting acquired non-susceptibility to at least one agent in three or more antimicrobial classes (3, 19). MDR infections persist even after curative interventions, including liver transplantation, and are linked to poorer post-transplant outcomes (13, 14).

Globally, MDRO prevalence among cirrhotic patients is approximately 34%, with the highest rates reported in Asia (particularly India) and South America (9). In Europe, MDRO prevalence in culture-positive infections among cirrhotic patients increased from 29% to 38% between 2011 and 2018 (20). A large multicenter intercontinental study highlighted geographic variability, with MDRO and extensively drug-resistant (XDR) organisms accounting for 73% and 33% of isolates, respectively, on the Indian subcontinent, compared with lower rates in North America (9). MDRO infections were associated with higher rates of septic shock, intensive care unit admission, prolonged hospitalization, and 28-day mortality (9).

Cirrhotic patients may be particularly susceptible to MDR infections due to repeated infections, frequent exposure to broad-spectrum antibiotics, recurrent hospitalizations, and invasive procedures. Approximately 37% of cirrhotic patients are readmitted within 30 days, potentially increasing exposure to healthcare-associated pathogens, including MDR strains (12, 21). Clinical and healthcare-related factors previously implicated as predictors of MDR infection include advanced liver disease, prior antibiotic exposure, healthcare-associated infections, intensive care unit admission, invasive procedures, and recurrent hospitalizations (22).

Among bacterial complications, spontaneous bacterial peritonitis (SBP) is the most common, affecting nearly one-third of cirrhotic patients, followed by urinary tract infections and pneumonia (9–11). Less frequent but severe complications, such as spontaneous bacteremia and bacterial empyema, are associated with high short-term mortality (9–11, 19). The pathogenesis of bacterial infections in cirrhosis is associated with intestinal barrier dysfunction, bacterial overgrowth, immune dysregulation, and bacterial translocation from the gut (10, 11, 15). Gram-negative enteric organisms, particularly Enterobacterales such as *Escherichia coli* and *Klebsiella pneumoniae*, are predominant, although Gram-positive pathogens, including *Enterococcus* and *Staphylococcus* species, also contribute substantially (10, 11, 15). Environmental, industrial, and agricultural factors especially in regions with limited regulation further contribute to the dissemination of resistance genes (23–29).

Early initiation of empiric antibiotic therapy is recommended in cirrhotic patients with infections such as SBP, pneumonia, and sepsis; delayed therapy is associated with higher hospital mortality (30). However, reliance on broad empirical regimens has contributed to antimicrobial overuse and increased resistance, with up to 45% of SBP cases showing resistance to first-line therapy (31). Given delays in culture-based susceptibility testing and regional variability in pathogen distribution, identifying local predictors of AMR is critical to guide empirical therapy and support antimicrobial stewardship (32). Despite the high burden of MDR infections in CLD, real-world, institution-specific data on predictors and local susceptibility patterns remain limited, impeding risk stratification and evidence-based management.

Based on this context, we hypothesized that healthcare exposure, prior antibiotic use, and Gram-negative infections would be associated with MDR infections in this population. This study therefore aimed to generate real-world evidence on the clinical, demographic, and healthcare-related predictors of antimicrobial resistance and to characterize local pathogen distribution and resistance patterns among patients with CLD in a tertiary care setting.

## 2 Methods

### 2.1 Study design and setting

This retrospective observational cohort study was conducted from February 2024 to August 2024 in the Department of Gastroenterology at a tertiary care hospital in Coimbatore, India.

### 2.2 Study population, eligibility criteria, and sample size

The study population included adult patients (≥18 years) diagnosed with CLD, including liver cirrhosis, liver fibrosis, and chronic hepatitis, who attended the Department of Gastroenterology during the study period. Both inpatient and outpatient cases with culture-positive bacterial infections were considered.

Exclusion criteria were: age < 18 years, pregnancy or lactation, fungal or viral infections, colonization samples without clinical evidence of infection, and duplicate bacterial isolates from the same patient. During the study period, 340 patients were assessed for eligibility. Of these, 23 were excluded: viral or fungal infections (*n* = 9), colonization without clinical evidence of infection (*n* = 6), and duplicate bacterial isolates (*n* = 8). A total of 317 patients met the inclusion criteria and were included in the final analysis.

### 2.3 Clinical specimens and microbiological methods

Clinical specimens including blood, ascitic fluid, urine, and other relevant samples were collected during routine clinical care based on the suspected site of infection. Samples were processed in the hospital microbiology laboratory using standard techniques. Specimens were cultured on appropriate media such as blood agar, MacConkey agar, and chocolate agar, and incubated under standard laboratory conditions. Bacterial isolates were identified using conventional biochemical tests and automated identification systems where available.

Antimicrobial susceptibility testing was performed using the Kirby–Bauer disc diffusion method and/or minimum inhibitory concentration (MIC) determination according to the Clinical and Laboratory Standards Institute (CLSI) guidelines (M100, 2022 edition). Results were interpreted as susceptible (S), intermediate (I), or resistant (R) based on established CLSI breakpoints. Resistance phenotypes, including extended-spectrum β-lactamase (ESBL) production and MDR, were determined according to CLSI criteria. Relevant microbiology data were retrospectively extracted from hospital laboratory records for analysis.

### 2.4 Data management and WHONET analysis

Microbiological data were entered into WHONET software version 5.6, a laboratory data management system developed by the World Health Organization Collaborating Center for Surveillance of Antimicrobial Resistance. WHONET was used to manage antimicrobial susceptibility data, generate institutional antibiograms, and calculate resistance percentages. Where applicable, the software supported analysis of trends in antibiotic resistance.

The software was configured according to CLSI interpretive criteria (M100, 2022 edition) to define antibiotic panels and breakpoints. Data analysis within WHONET included selection of bacterial species, specimen types, and antibiotics of interest, with calculation of MIC50, MIC90, and resistance trends. Resistance phenotypes such as MRSA, ESBL, and AmpC β-lactamase production were also analyzed where applicable.

### 2.5 Statistical analysis

All statistical analyses were performed using SPSS version 28.0 (IBM Corp., Armonk, NY, USA). Categorical variables (e.g., gender, infection type, specimen distribution, and microbiological profiles) were summarized as frequencies and percentages. Continuous variables, such as age, were assessed for normality and presented as mean ± standard deviation (SD) or median with interquartile range (IQR), as appropriate. Group comparisons were conducted using the Chi-square (χ^2^) test for categorical variables to evaluate associations between demographic, clinical, and microbiological characteristics. For non-normally distributed continuous variables, the Mann–Whitney *U*-test was applied. Organism-specific MDR rates were calculated. Odds ratios (ORs) with 95% confidence intervals (CIs) were estimated to assess the association between individual pathogens and MDR, using *Staphylococcus aureus* as the reference category. Additionally, the comparative resistance burden between Gram-negative and Gram-positive organisms was evaluated using relative risk (RR) with corresponding 95% CIs.

Institutional antibiograms were constructed using WHONET-derived data and interpreted according to CLSI M100 (2022) guidelines. Antimicrobial susceptibility patterns were expressed as percentages with 95% CIs. Minimum inhibitory concentration (MIC) distributions were summarized using MIC50 and MIC90 values. Resistance trends across antibiotic classes and the prevalence of ESBL production were also evaluated, with trend analysis performed where applicable. To identify predictors of MDR infection, univariable logistic regression analysis was initially performed. Variables with *p* < 0.10 were included in a multivariable logistic regression model. Adjusted odds ratios (aORs) with 95% CIs were reported to determine independent associations, including age, gender, infection type, organism category, and prior antibiotic exposure. All statistical tests were two-tailed, and a *p*-value < 0.05 was considered statistically significant.

### 2.6 Ethical approval

The study protocol was reviewed and approved by the Institutional Human Ethics Committee (IHEC), PSG Institute of Medical Sciences and Research (Approval No: PSG/IHEC/2024/Appr/Exp/057; Project No: 24/009). The requirement for informed consent was waived due to the retrospective nature of the study.

## 3 Results

### 3.1 Baseline characteristics

A total of 317 patients with CLD were included. The mean age was 50.9 ± 10.4 years (median 50, IQR 44–59), with the majority aged 41–50 years, followed by 61–70 and 51–60 years. The cohort was predominantly male (83.9%). Single-organism infections were more frequent than multiple-organism infections (76.0% vs. 24.0%), and community-acquired infections were more common than healthcare-associated infections (56.5% vs. 43.5%) (Table 1).

**Table 1:** Demographic and clinical characteristics of patients with chronic liver disease (*n* = 317).

Variable	Category	*n* (%)	*p*-value
**Age group**	≤ 40	46 (14.5)	<0.001
	41–50	89 (28.1)	
	51–60	76 (24.0)	
	61–70	85 (26.8)	
	≥71	21 (6.6)	
**Age (years)**	Mean SD	50.9 ± 10.4	
	Median (IQR)	50 (44–59)	
**Gender**	Male	266 (83.9)	0.018
	Female	51 (16.1)	
**Specimen positivity**	Single isolate	241 (76.0)	0.042
	Multiple isolates	76 (24.0)	
**Infection type**	Community-acquired	179 (56.5)	<0.001
	Healthcare-associated	138 (43.5)	

Chi-square test for categorical variables, Mann–Whitney *U*-test for age.

### 3.2 Infection profile

Urine was the most common specimen source (29.7%), followed by blood (25.2%) and ascitic fluid (20.8%), while respiratory samples were least frequent (5.4%) (Table 2). Infections were predominantly observed in male patients across all specimen types (76.6%−88.2%; *p* < 0.05). Healthcare-associated infections were more common in respiratory (70.6%) and wound/pus samples (65.7%), followed by blood (52.5%) and ascitic fluid (48.4%), whereas urine samples showed a lower proportion (41.5%). Differences in infection acquisition across specimen types were statistically significant (*p* = 0.002–0.031).

**Table 2:** Specimen distribution stratified by infection acquisition and gender.

Specimen type	Total *n* (%)	Male *n* (%)	Female *n* (%)	Healthcare-associated %	*p*-value
Urine	94 (29.7)	72 (76.6)	22 (23.4)	41.5	0.031
Blood	80 (25.2)	69 (86.3)	11 (13.7)	52.5	0.002
Ascitic fluid	66 (20.8)	58 (87.9)	8 (12.1)	48.4	0.009
Wound/Pus	35 (11.0)	27 (77.1)	8 (22.9)	65.7	0.018
Respiratory	17 (5.4)	15 (88.2)	2 (11.8)	70.6	0.006

### 3.3 Distribution of pathogens and MDR risk

The most frequently isolated organisms were *Escherichia coli* (37.5%), *Klebsiella pneumoniae* (14.5%), and *Enterococcus faecium* (9.1%) (Table 3). MDR prevalence was notably high among Gram-negative organisms, including *K. pneumoniae* (82.6%) and *E. coli* (78.2%). Relative to *Staphylococcus aureus, E. coli* (OR 2.41; 95% CI 1.45–4.01; *p* < 0.001), *K. pneumoniae* (OR 3.12; 95% CI 1.58–6.15; *p* < 0.001), and *E. faecium* (OR 1.98; 95% CI 1.02–3.87; *p* = 0.041) showed significantly higher odds of MDR, whereas *Pseudomonas aeruginosa* was not significantly associated (OR 1.64; 95% CI 0.81–3.33; *p* = 0.161).

**Table 3:** Organism distribution with resistance risk metrics.

Organism	*n* (%)	MDR %	Odds ratio for MDR (95% CI)	*p*-value
*Escherichia coli*	119 (37.5)	78.2	2.41 (1.45–4.01)	<0.001
*Klebsiella pneumoniae*	46 (14.5)	82.6	3.12 (1.58–6.15)	<0.001
*Enterococcus faecium*	29 (9.1)	69.0	1.98 (1.02–3.87)	0.041
*Pseudomonas aeruginosa*	27 (8.5)	63.0	1.64 (0.81–3.33)	0.161
*Staphylococcus aureus*	23 (7.3)	47.8	Reference	—

MDR, Multidrug-resistant; CI, Confidence Interval.

### 3.4 Antimicrobial resistance patterns

MDR was more prevalent among Gram-negative isolates than Gram-positive isolates (70.2% vs. 51.9%), reflecting a significantly higher MDR risk in Gram-negative pathogens (RR 1.35; 95% CI 1.07–1.70; *p* = 0.003) (Table 4). Fluoroquinolone resistance was high in both groups, but significantly higher among Gram-positive isolates (87.3% vs. 74.8%; RR 0.86; 95% CI 0.77–0.97; *p* = 0.021). Carbapenem resistance occurred exclusively in Gram-negative isolates (50.0%).

**Table 4:** Comparative resistance burden between gram-negative and gram-positive isolates.

Resistance parameter	Gram-negative (*n* = 238)	Gram-positive (*n* = 79)	Relative risk (95% CI)	*p*-value
MDR phenotype	167 (70.2%)	41 (51.9%)	1.35 (1.07–1.70)	0.003
Fluoroquinolone resistance	178 (74.8%)	69 (87.3%)	0.86 (0.77–0.97)	0.021
Carbapenem resistance	119 (50.0%)	NA	—	—
Aminoglycoside resistance	104 (43.7%)	39 (49.4%)	0.88 (0.68–1.14)	0.337

CI, Confidence Interval.

### 3.5 Antibiogram analysis

Gram-positive isolates remained largely susceptible to glycopeptides (90.2%) and oxazolidinones (92.7%), with low MIC values and stable susceptibility trends (Table 5). Tetracyclines demonstrated complete susceptibility (100%), indicating improving resistance trends. In contrast, fluoroquinolones (12.7%) and β-lactam antibiotics (18.9%) exhibited very low susceptibility, associated with high MIC values and declining trends.

**Table 5:** Advanced institutional antibiogram for gram-positive isolates (CLSI-compliant).

Antibiotic class	Susceptibility % (95% CI)	MIC50	MIC90	Trend
Glycopeptides	90.2 (77.5–96.1)	0.5	1	Stable
Oxazolidinones	92.7 (80.6–97.5)	0.25	0.5	Stable
Tetracyclines	100 (95.4–100)	0.5	1	Improving
Fluoroquinolones	12.7 (6.6–22.8)	8	16	Declining
β-lactams	18.9 (11.2–30.0)	>32	>32	Declining

CI, -Confidence Interval; MIC, Minimum Inhibitory Concentration.

**Figure 1:**
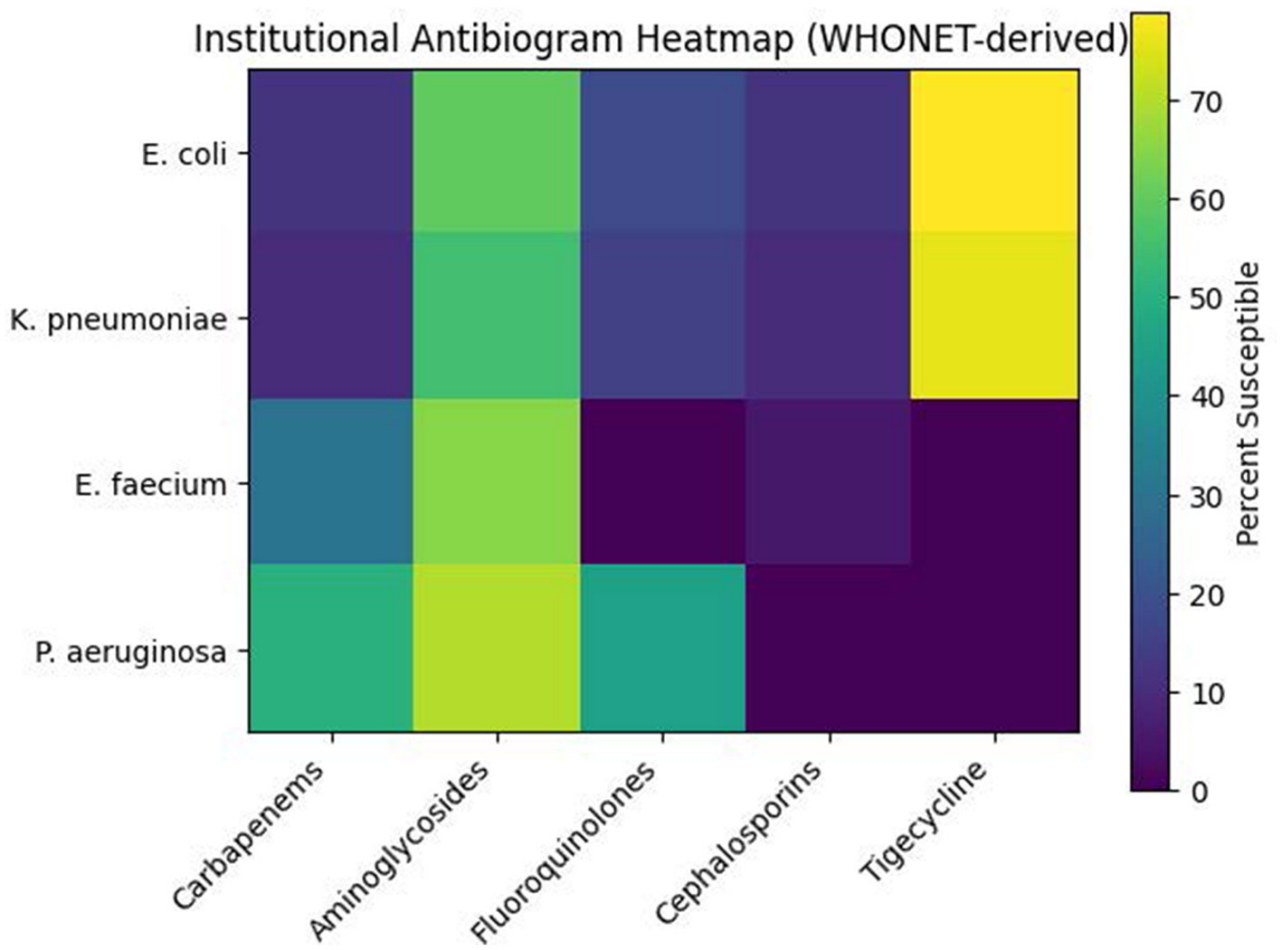
WHONET-derived institutional antibiogram heatmap showing antibiotic susceptibility patterns of clinically relevant gram-negative and enterococcal isolates. Color intensity represents percentage susceptibility derived from WHONET v5.6 analysis. Antibiotic susceptibility results were interpreted according to the guidelines of the Clinical and Laboratory Standards Institute (CLSI) M100 (2022) interpretive criteria. Rows represent clinically relevant organisms, and columns represent antibiotic classes. This map highlights marked resistance to cephalosporins and fluoroquinolones among gram-negative isolates, with relatively preserved susceptibility to tigecycline and aminoglycosides.

Among Gram-negative isolates, tigecycline showed the highest susceptibility (79.1%), followed by aminoglycosides with moderate retained activity (60.3%) (Table 6). Carbapenems demonstrated reduced susceptibility (49.8%), reflecting limited effectiveness. A substantial proportion of isolates were ESBL producers (42.5%−53.4%). Third-generation cephalosporins (12.1%) and fluoroquinolones (17.9%) exhibited very low susceptibility, associated with high ESBL prevalence and significantly declining resistance trends. These findings are consistent with the patterns shown in Figure 1.

**Table 6:** Advanced institutional antibiogram for gram-negative isolates.

Antibiotic class	Susceptibility %	MIC50	MIC90	ESBL %	*p*-trend
Tigecycline	79.1	1	2	NA	0.041
Aminoglycosides	60.3	4	8	42.5	0.019
Carbapenems	49.8	4	16	53.4	<0.001
3rd-gen cephalosporins	12.1	>32	>32	71.2	<0.001
Fluoroquinolones	17.9	8	16	64.8	<0.001

MIC, Minimum Inhibitory Concentration; ESBL, Extended Spectrum β-Lactamase.

### 3.6 Predictors of multidrug resistance

Multivariable logistic regression identified several independent predictors of MDR infection (Table 7). Age ≥ 60 years (adjusted OR 1.89; 95% CI 1.12–3.21; *p* = 0.017) and male gender (adjusted OR 1.67; 95% CI 1.01–2.77; *p* = 0.046) were independently associated with higher odds of MDR infection. Healthcare-associated infections conferred the highest risk, with more than a threefold increase (adjusted OR 3.14; 95% CI 1.92–5.14; *p* < 0.001). Infection with Gram-negative organisms (adjusted OR 2.73; 95% CI 1.61–4.64; *p* < 0.001) and prior antibiotic exposure (adjusted OR 2.98; 95% CI 1.74–5.10; *p* < 0.001) were also significant predictors.

**Table 7:** Multivariable predictors of multidrug-resistant infection.

Variable	Adjusted OR	95% CI	*p*-value
Age ≥ 60 years	1.89	1.12–3.21	0.017
Male gender	1.67	1.01–2.77	0.046
Healthcare-associated infection	3.14	1.92–5.14	<0.001
Gram-negative organism	2.73	1.61–4.64	<0.001
Prior antibiotic exposure	2.98	1.74–5.10	<0.001

OR, Odds ratio; CI, Confidence Interval.

## 4 Discussion

Bacterial infections remain one of the most important drivers of morbidity and mortality in patients with CLD and cirrhosis, and their intersection with antimicrobial resistance represents an increasingly critical clinical challenge. This study adds to the growing body of real-world evidence demonstrating that MDR infections are now highly prevalent and clinically significant in this vulnerable population. In our cohort of 317 patients, MDR prevalence was substantial, affecting 70.2% of Gram-negative isolates and 51.9% of Gram-positive isolates, indicating a markedly higher risk associated among Gram-negative organisms (35% relative increase). These findings indicate that more than half of culture-positive infections in patients with CLD are caused by MDR pathogens, underscoring the severity of the resistance burden in contemporary clinical practice.

The predominance of Gram-negative organisms, particularly Enterobacterales such as *Escherichia coli* (37.5%) and *Klebsiella pneumoniae* (14.5%), is consistent with well-established pathophysiological mechanisms in cirrhosis, including intestinal dysbiosis, increased gut permeability, and bacterial translocation (5, 12). These processes may facilitate systemic dissemination of enteric bacteria (7) and explain why Gram-negative pathogens continue to dominate infection profiles among cirrhotic patients across diverse geographical settings (8). The high burden of MDR observed among these organisms in our cohort reflects a broader global trend of escalating resistance to β-lactams, fluoroquinolones, and carbapenems, particularly in healthcare-associated infections (4, 14).

Healthcare exposure is strongly associated with MDR infections, highlighting the role of nosocomial transmission and antibiotic selection pressure in shaping resistance patterns. In our study, independent predictors included healthcare-associated infections, older age, male gender, Gram-negative organisms, and prior antibiotic exposure. Hospital-acquired infections often involve MDR organisms, with repeated hospitalizations, invasive procedures, and prior antibiotic use creating conditions that favor resistant strains. Surveillance studies show that MDR infections are more common in hospital settings than in the community, emphasizing the need for effective infection control and antibiotic stewardship (33, 34). These findings are consistent with large multicentre studies in cirrhotic populations, which have similarly demonstrated that MDR infections are more closely linked to healthcare contact than to community-driven transmission (9, 13).

Advanced liver disease further amplifies susceptibility to MDR infections. Patients with decompensated cirrhosis exhibit profound immune dysfunction, characterized by impaired neutrophil activity, reduced complement levels, and dysregulated cytokine responses collectively described as cirrhosis-associated immune dysfunction (CAID) (35, 36). This immunocompromised state, particularly in patients with prior antibiotic exposure or hepatic encephalopathy, increases susceptibility to infection and may facilitate colonization and persistence of multidrug-resistant organisms (37, 38). In our study, prior antibiotic use emerged as a strong independent predictor of MDR infection, reinforcing the central role of antibiotic pressure in resistance development.

Demographic factors were also independently associated with MDR risk. Older age (≥60 years) and male gender were independently associated with a higher likelihood of MDR infection. In older patients, age-related immunosenescence, comorbidities, and cumulative antibiotic exposure contribute to increased susceptibility to infections and multidrug-resistant organisms (37, 39, 40). Although the mechanisms underlying gender differences in MDR risk remain incompletely understood, variations in healthcare-seeking behavior, occupational exposure, comorbidity profiles, and microbiome composition have been proposed as potential contributing factors (41).

Importantly, while predictors of AMR in patients with chronic liver disease have been described previously, our study provides several novel insights. This real-world cohort of 317 patients from a tertiary care center in South India included both community-acquired and healthcare-associated infections, allowing a direct comparison of MDR risk across infection settings. Using WHONET-based analyses, we comprehensively characterized pathogen-specific resistance patterns, temporal trends, and ESBL prevalence, providing detailed, locally relevant data to inform antimicrobial stewardship. Furthermore, we quantified the independent contributions of older age, male gender, Gram-negative etiology, healthcare-associated acquisition, and prior antibiotic exposure to MDR risk, offering precise risk estimates to guide empiric therapy and targeted infection control strategies. These findings extend previous reports by providing clinically actionable insights into MDR epidemiology and therapeutic considerations in contemporary CLD care.

The antimicrobial susceptibility patterns observed in this study have important therapeutic implications. Tigecycline and aminoglycosides retained relatively higher activity against Gram-negative pathogens, consistent with recent surveillance data (42, 43). However, their use in patients with advanced liver disease must be carefully balanced against potential toxicity and organ dysfunction. In contrast, the markedly reduced susceptibility to carbapenems and third-generation cephalosporins is particularly concerning, as these agents have traditionally formed the backbone of empirical therapy for severe infections in cirrhosis (14, 44). The observed decline in carbapenem susceptibility, together with the high prevalence of ESBL-producing organisms, underscores the urgent need for institution-specific antibiograms and risk-stratified empirical treatment strategies rather than reliance on generalized treatment guidelines.

Beyond antimicrobial selection, MDR infections also have significant prognostic implications. Previous studies have consistently demonstrated that MDR infections in cirrhotic patients are associated with adverse clinical outcomes, including septic shock, acute-on-chronic liver failure (ACLF), increased intensive care unit admission, and higher short-term mortality (11, 19). These observations highlight that antimicrobial resistance is not merely a microbiological concern but a critical determinant of disease trajectory and survival in patients with CLD.

Early risk stratification of patients based on healthcare exposure, prior antibiotic use, infecting organism, and infection source is therefore essential to guide timely initiation of appropriate empirical therapy and improve clinical outcomes (10, 45). In this context, the identification of Gram-negative infection and healthcare-associated acquisition as strong independent predictors of MDR provides actionable insights for clinical decision-making.

Finally, the findings of this study support the growing call for strengthened antimicrobial stewardship programs tailored specifically to hepatology and transplant patient populations. Such programs should integrate real-time microbiological surveillance, clinician education, and multidisciplinary decision-making to optimize antibiotic use while minimizing resistance selection pressure (1, 46). In parallel, robust infection prevention strategies including hand hygiene, environmental decontamination, and targeted screening for MDR colonization are critical to curbing nosocomial transmission in high-risk settings (2, 47). A combination of improved surveillance, individualized risk assessment, and judicious antibiotic use will be essential to mitigate the growing challenge of MDR infections and improve outcomes in this highly vulnerable patient population.

### 4.1 Implications for clinical practice

#### 4.1.1 Empirical antibiotic therapy

Recognition of key risk factors identified in this study including healthcare-associated acquisition, Gram-negative infections, older age, and prior antibiotic exposure can guide the selection of initial empiric antibiotics. Tailoring therapy to these risk profiles may reduce delays in effective treatment, limit exposure to unnecessary broad-spectrum agents, and improve patient outcomes.

#### 4.1.2 Antimicrobial stewardship strategies

Implementation of institution-specific surveillance, real-time microbiological monitoring, and targeted clinician education is essential to minimize inappropriate antibiotic use and prevent further emergence of multidrug-resistant organisms. Regular updates to local antibiograms based on real-world data can support evidence-based empiric therapy and inform stewardship policies.

#### 4.1.3 Risk stratification

Early identification of high-risk patients enables proactive infection prevention, focused monitoring, and individualized treatment plans. Stratifying patients according to infection source, organism type, and prior healthcare exposure allows clinicians to prioritize interventions for those most vulnerable, ultimately improving clinical outcomes and reducing morbidity and mortality in patients with chronic liver disease.

### 4.2 Strengths and limitations

This study has several strengths, including a well-defined cohort of patients with chronic liver disease, standardized microbiological methods, comprehensive antimicrobial susceptibility testing using WHONET software, and robust statistical analyses to identify independent predictors of multidrug resistance. The inclusion of both community-acquired and healthcare-associated infections provides a representative overview of the infection burden in this population.

However, several limitations should be acknowledged. The retrospective design precludes causal inference and may be subject to selection bias. As a single-center study, findings may not be generalizable to regions with differing resistance patterns or antimicrobial prescribing practices. Inclusion was limited to culture-positive infections, which may underestimate the overall infection burden. Data on liver disease etiology, severity indices (Child-Pugh and MELD scores), and detailed antibiotic exposure histories were not uniformly available, restricting more granular risk stratification. Additionally, clinical outcomes such as mortality, treatment response, and length of hospital stay were not assessed. The relatively short study duration may also limit evaluation of long-term resistance trends.

## 5 Conclusion

In this real-world study of patients with CLD, multidrug-resistant infections were prevalent, particularly among Gram-negative pathogens such as *Escherichia coli* and *Klebsiella pneumoniae*. Resistance patterns varied by specimen type, infection setting, and pathogen, with healthcare-associated infections exhibiting higher MDR rates. While many Gram-positive isolates remained susceptible to glycopeptides, oxazolidinones, and tetracyclines, resistance to fluoroquinolones and β-lactams was notable. Among Gram-negative isolates, tigecycline and aminoglycosides retained moderate activity, whereas carbapenems, third-generation cephalosporins, and fluoroquinolones showed reduced effectiveness.

Older age, male gender, healthcare-associated infection, Gram-negative etiology, and prior antibiotic exposure were independently associated with a higher likelihood of MDR infection. These findings emphasize the importance of informed antibiotic selection, ongoing infection surveillance, and targeted antimicrobial stewardship to optimize care and outcomes in patients with CLD.

### 5.1 Future directions

Future research should prioritize prospective, multicenter studies that integrate detailed clinical outcomes, liver disease severity indices, and longitudinal antibiotic exposure data. Additionally, studies should evaluate the cost-effectiveness of empiric antibiotic strategies and their influence on hospital length of stay and mortality. Exploration of novel diagnostic approaches, alternative therapeutic agents, and optimized combination regimens may help mitigate the growing burden of MDR infections in patients with chronic liver disease. Finally, assessing the effectiveness of antimicrobial stewardship interventions specifically tailored to hepatology populations remains an urgent research priority.

## Data Availability

The original contributions presented in the study are included in the article/supplementary material, further inquiries can be directed to the corresponding author.
